# Durable Improvement in Generic and Fibroid-Specific Quality of Life in Women Treated with Transcervical Fibroid Ablation with the Sonata System After Three Years

**DOI:** 10.1089/gyn.2021.0073

**Published:** 2022-04-01

**Authors:** Kelly Roy, James K. Robinson

**Affiliations:** ^1^ Arizona Gynecology Consultants, Phoenix, Arizona, USA.; ^2^ MedStar Washington Hospital Center, Washington, District of Columbia, USA.

**Keywords:** leiomyoma, quality-adjusted life year, quality of life, QALY, transcervical fibroid ablation, uterine fibroid

## Abstract

**Objective:**

To determine quality-adjusted life years (QALYs) over 3 years after transcervical fibroid ablation (TFA) with the Sonata System.

**Methods:**

The SONATA trial was a prospective multicenter interventional trial that assessed the safety and efficacy of TFA for treatment of women with symptomatic uterine fibroids. Change in generic health status was assessed with the EuroQol 5-Dimension questionnaire (0–1 scale). Fibroid-specific quality of life (QOL) was measured on a 0 to 100 scale with the health-related quality of life subscale of the Uterine Fibroid Symptom and Quality-of-Life (UFS-QOL). The number of QALYs gained relative to baseline and cumulative QALYs were calculated using the area under the curve at each follow-up visit over 3 years.

**Results:**

Among 147 women receiving TFA, fibroid-specific QOL increased from 40 ± 21 at baseline to 84 ± 19 at 1 year and 83 ± 23 at 3 years (*p* < 0.001). Generic QOL increased from 0.72 ± 0.21 at baseline to 0.89 ± 0.12 at 1 year and 0.88 ± 0.16 at 3 years (*p* < 0.001). Over 3 years, TFA resulted in 1.24 ± 0.64 QALYs gained when using fibroid-specific health utility scores and 0.49 ± 0.61 QALYs gained when using generic health utility scores. Cumulative QALYs experienced at 3 years as a percentage of perfect health were 82% with fibroid-specific scores and 88% with generic health scores.

**Conclusions:**

Women treated by TFA with the Sonata System for symptomatic uterine fibroids reported durable improvements in generic and fibroid-specific QOL, as well as clinically meaningful increases in QALYs over 3 years. Clinical Trials.gov ID: NCT02228174. (J GYNECOL SURG 38:143)

## Introduction

Uterine fibroids are benign tumors often presenting clinically with symptoms that include abnormal uterine bleeding, pelvic pressure, and subfertility.^1^ Although uterine fibroids may occur at any time during the reproductive years and have a peak incidence before menopause, fibroids and their associated symptoms occasionally persist into menopause.^2^ The societal impact of symptomatic uterine fibroids is often under-recognized along with the marked reduction in quality of life (QOL) among affected women.^1^ QOL is an important metric in women suffering from uterine fibroids, since derivations of this measurement can be used to assist in decision-making regarding prioritization of health care resources. For example, adoption of a uterine fibroid treatment that reduces fibroid volume and associated symptoms may be rational if a concomitant improvement in self-reported QOL was observed. An idealized uterine fibroid treatment would safely and effectively reduce fibroid size, durably relieve associated symptoms, and result in sustained QOL improvements.

Transcervical fibroid ablation (TFA) is a minimally invasive procedure that has emerged as a safe and effective alternative to hysterectomy.^3–6^ Long-term results with TFA utilizing the Sonata System demonstrate durable symptom improvement with low surgical reintervention rates (8.2% at 36 months). Health outcomes after TFA, including QOL data, health utility, and quality-adjusted life years (QALYs), have been reported through 1 year post-treatment.^7^ However, longer term changes in health utility and the corresponding QALYs have not been characterized. Using data from the Sonography Guided Transcervical Ablation of Uterine Fibroids (SONATA) trial, the purpose of this study was to determine the health outcomes of TFA utilizing the Sonata System on QOL as determined by QALYs through over 3 years of follow-up.

## Methods

The SONATA trial was a prospective multicenter interventional trial that was designed to assess the safety and efficacy of TFA for treatment of symptomatic uterine fibroids (ClinicalTrials.gov NCT02228174). The clinical trial was conducted according to the principles of the Helsinki Declaration, participating centers received institutional review board or ethics committee approval (Table 1), and all patients provided written informed consent before trial participation. Eligible patients were premenopausal women between 25 and 50 years with heavy menstrual bleeding associated with uterine fibroids. A complete list of trial eligibility criteria and clinical outcomes through 3 years are available elsewhere.^8–10^ Three-year clinical outcomes from this trial have been previously reported. This is the first report of long-term healthy utility results after TFA in the SONATA trial.

**Table 1.** List of Participating Sites and Institutional Review Boards/Ethics Committees

Institution name	IRB/ethics committee
Arizona Gynecology Consultants	Western IRB
Advanced Women's Health Institute	Western IRB
Universidad Autónoma de Nuevo Leon, Monterrey	Facultad de Medicina y Hospital Universitario
GW Medical Faculty Associates	Western IRB
Christiana Care Health System	Christiana Care IRB
KO Clinical Research	Western IRB
Virtus Research Consultants	Western IRB
University of Maryland School of Medicine	University of Maryland-Baltimore IRB
Wayne State University Physician Group	Western IRB
Mercy Clinic	Western IRB
University of Mississippi Medical Center	University of Mississippi Medical Center IRB
Women's Wellness Clinic	Western IRB
Cooper University Hospital	Cooper Health System IRB
Bosque Women's Care	Western IRB
Albert Einstein School of Medicine-Montefiore	Biomedical Research Alliance of New York IRB
Drexel University College of Medicine	Western IRB
Magee-Women's Hospital	Western IRB
PRISMA Health Upstate	Health Sciences South Carolina IRB
Baylor Research Institute	Baylor Research Institute IRB
Willowbend Health and Wellness	Western IRB
Eastern Virginia Medical School	Eastern Virginia Medical School IRB
Virginia Mason Medical Center	Western IRB

IRB, Institutional Review Board.

TFA device characteristics and procedural details have been described in detail elsewhere.^8–10^ The procedure utilizes the Sonata^®^ system (Gynesonics, Inc., Redwood City, CA), which consists of an integrated intrauterine sonography probe and radiofrequency ablation handpiece that facilitates fibroid identification, targeting, and ablation. A graphical interface is displayed on a live ultrasound image that identifies the target ablation area and the extent of subablative thermal heating. This information is used to confirm the ablation size and location while confining the thermal safety border to within the uterine serosa.

After the procedure, patients returned for clinical follow-up at regular intervals for 3 years. Change in generic health status was assessed with the EuroQol 5-Dimension (EQ-5D) questionnaire, which comprises the five dimensions of mobility, self-care, usual activities, pain/discomfort, and anxiety/depression.^11^ Utility scores derived from the EQ-5D range from 0 (death) to 1 (perfect health). Fibroid-specific QOL was measured using the health-related quality of life (HRQL) subscale of the Uterine Fibroid Symptom and Quality-of-Life (UFS-QOL) Questionnaire, with scores ranging from 0 (worst QOL) to 100 (best QOL).^12^ Values for HRQL and EQ-5D were additionally reported using standardized minimal clinically important difference (MCID) units, defined as the mean change from baseline divided by the MCID.^13,14^ A 20-point increase from baseline for HRQL^12^ and a 0.074-point increase from baseline for EQ-5D^15^ are considered clinically important improvements. An improvement from baseline of <0.5 MCID units suggests that it is unlikely that an appreciable number of patients will show a clinically important benefit, improvements between 0.5 and 1 MCID units suggest that treatment may benefit an appreciable number of patients, and improvements >1 MCID unit indicate that many patients may gain important benefits from treatment.^13,14^

We determined the number of QALYs gained relative to baseline by calculating the area under the curve at each follow-up visit using the trapezoidal rule to account for serial measurements.^16^ We additionally determined cumulative QALYs by multiplying the mean health utility score by the duration of follow-up in years. One QALY represents 1 year in perfect health, death is assigned 0 QALYs, and some health states are considered worse than death and associated with negative scores.^17,18^ A last observation carried forward sensitivity analysis was performed whereby missing follow-up data were imputed with the most recent score. Data were analyzed using SAS version 9.3 (SAS Institute, Cary, NC). Subjects served as their own control for all pre- to postanalyses, statistical tests comparing 3-year results with baseline were two-sided, and *p*-values <0.05 indicated a statistically significant change.

## Results

The SONATA trial enrolled 147 women at 22 centers who all received TFA with the Sonata System for symptomatic uterine fibroids. Participants were enrolled at a mean 43 years of age and received TFA to ablate an average of 3.0 ± 2.1 fibroids per patient, where the average treated fibroid diameter was 2.5 ± 1.2 cm. Generic health status at baseline measured on the EQ-5D was 0.72, considerably below the age-matched population mean of 0.87.^19^

**FIG. 1.**
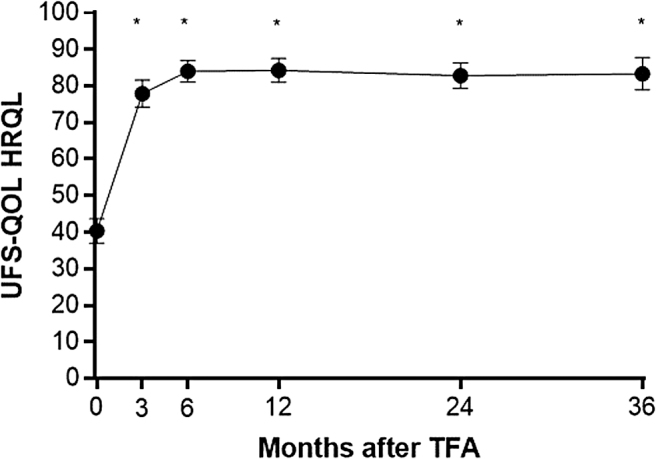
Change in HRQL subscale of the UFS-QOL Questionnaire over 3 years after TFA. Plotted values are mean and 95% confidence interval. *Asterisk* indicates statistically significant change relative to baseline (*p* < 0.05). HRQL, health-related quality of life; QOL, quality of life; TFA, transcervical fibroid ablation; UFS-QOL, Uterine Fibroid Symptom and Quality-of-Life.

**FIG. 2.**
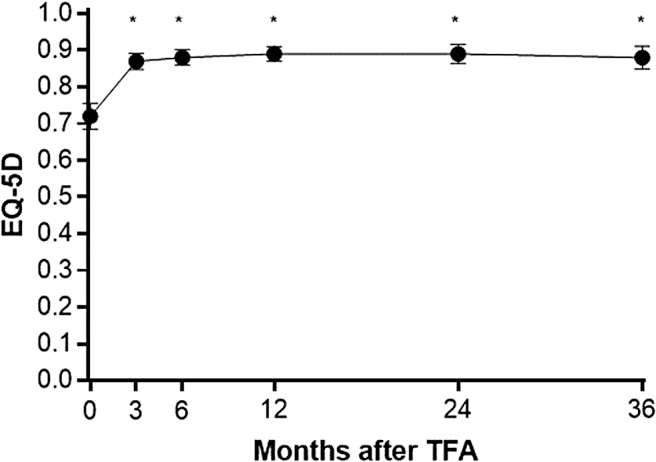
Change in EQ-5D questionnaire score over 3 years after TFA. Plotted values are mean and 95% confidence interval. *Asterisk* indicates statistically significant change relative to baseline (*p* < 0.05). EQ-5D, EuroQol 5-Dimension.

**FIG. 3.**
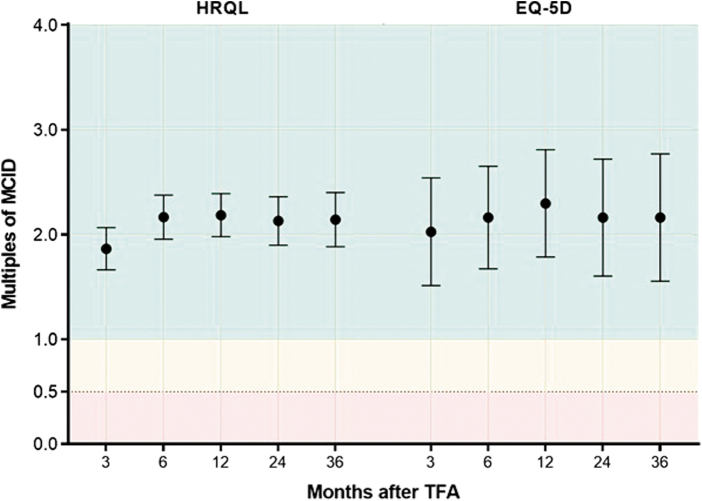
Improvement in QOL from baseline after TFA reported in standardized MCID units with 95% confidence intervals. The MCID is 20 points for HRQL and 0.074 points for EQ-5D questionnaire. Treatment effects <0.5 MCID units (denoted by *red* background) indicate that it is unlikely that an appreciable number of patients will show a clinically important benefit, treatment effects between 0.5 and 1 MCID units (denoted by *yellow* background) indicate that a treatment may be beneficial to an appreciable number of patients, and treatment effects >1 MCID unit (denoted by *green* background) indicate that many patients may gain important benefits from treatment.^13,14^ MCID, minimal clinically important difference.

**FIG. 4.**
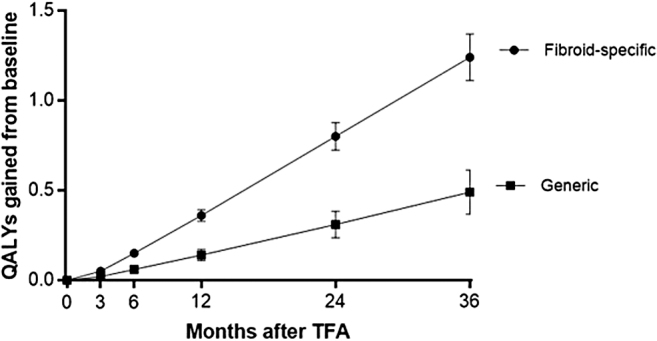
QALYs gained relative to baseline over 3 years after TFA. Plotted values are mean and 95% confidence interval. QALYs gained from baseline were calculated for generic QOL using the EQ-5D questionnaire and fibroid-specific QOL using the HRQL subscale of the UFS-QOL questionnaire. QALYs, quality-adjusted life years.

**FIG. 5.**
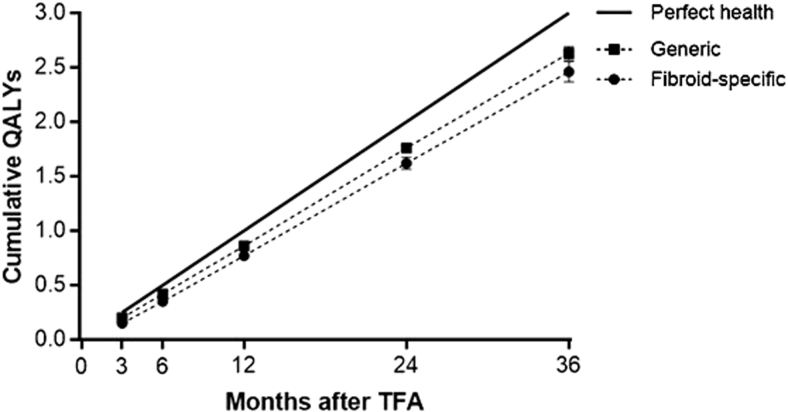
Cumulative QALYs over 3 years after TFA. Plotted values are mean and 95% confidence interval. Cumulative QALYs were calculated for generic QOL using the EQ-5D questionnaire and fibroid-specific QOL using the HRQL subscale of the UFS-QOL questionnaire.

Patient adherence during follow-up was favorable with 90% accounted for at 3 years. The rate of surgical reintervention for heavy menstrual bleeding was 0.7% at 1 year, 5.5% at 2 years, and 9.2% at 3 years. Fibroid-specific QOL increased from 40 ± 21 at baseline to 84 ± 19 at 1 year and 83 ± 23 at 3 years (*p* < 0.001) (Fig. 1). Generic QOL increased from 0.72 ± 0.21 at baseline to 0.89 ± 0.12 at 1 year and 0.88 ± 0.16 at 3 years (*p* < 0.001) (Fig. 2). When considering these treatment effects associated with TFA at each follow-up visit over 3 years in relation to established MCIDs, the improvements ranged from 1.9 to 2.2 MCID units for fibroid-specific QOL and 2.0 to 2.3 MCID units for generic QOL (Fig. 3), indicating that for each of these outcomes at each follow-up interval, “many patients may gain important benefits from treatment.”^13,14^ Comparing outcomes at 3 years relative to baseline, TFA resulted in 1.24 ± 0.64 QALYs gained when calculated using fibroid-specific health utility scores and 0.49 ± 0.61 QALYs gained when calculated using generic health utility scores (Fig. 4). QALYs experienced at 3 years post-TFA with the Sonata System relative to perfect health were 82% when using fibroid-specific scores and 88% when using generic health scores (Fig. 5). Results of the last observation carried forward sensitivity analysis corroborated the findings of the primary analysis with 1.18 ± 0.66 QALYs gained based on fibroid-specific health utility scores and 0.47 ± 0.59 QALYs gained using generic health utility scores.

## Discussion

In women with symptomatic uterine fibroids, TFA with the Sonata System provided clinically meaningful and durable improvements in generic and fibroid-specific QOL. These improvements were detected as early as 3 months postablation, continued to improve throughout the remainder of the 3-year follow-up period, and were twofold greater than established MCID thresholds for both generic and fibroid-specific QOL. The magnitude of these improvements indicates that clinically important treatment benefits were realized in most patients receiving TFA. This study represents the longest follow-up period during which QALY was assessed after TFA. The results of this study have important implications for women and their providers when engaging in shared decision-making to identify their optimal treatment for uterine fibroids.

In a previous study that evaluated TFA with the Sonata System through 12 months in a 50-patient cohort of women with symptomatic uterine fibroids,^7^ patients experienced 0.21 QALYs at 3 months, 0.43 QALYs at 6 months, and 0.91 QALYs at 1 year. This study reports comparable values (0.20, 0.42, and 0.86 QALYs at 3, 6, and 12 months, respectively), but extends these findings through 3 years of follow-up and demonstrates that health utility is maintained through 36 months after TFA. The magnitudes of generic and fibroid-specific QOL were clinically important and demonstrate treatment durability after TFA in the treated cohort through 3 years. QALYs experienced by patients treated with TFA are comparable with those reported after treatments such as hysterectomy, uterine artery embolization, and myomectomy.^21^ TFA resulted in ∼0.41 QALYs gained per year using fibroid-specific health utility scores and 0.16 QALYs gained per year using generic health utility scores) over 3 years of follow-up in this study. For reference, typical QALYS gained per year with other highly effective therapies are 0.25 for hip arthroplasty,^22^ 0.17 for knee arthroplasty,^22^ 0.09 for continuous positive airway pressure,^23^ and 0.04 for coronary artery bypass grafting.^24^ TFA presents as an attractive alternative for treatment of uterine fibroids given the significantly lower cost compared with transperitoneal hysterectomy and myomectomy,^25^ the less invasive incisionless uterus preserving approach, significant safety and effectiveness outcomes at 1 year that are maintained through 3 years, and clinically important QALYs gained at 3 years.^8,10^

The primary strengths of this study are a large sample size of patients with wide geographical representation and long-term patient follow-up. A limitation of the study is that QOL was determined by the answers to patient-reported questionnaires that could be influenced by expectation bias among patients. Also, health care costs were not a component of this study; however, the results could be used to facilitate future health economic studies such as cost-effectiveness and/or cost-utility analyses.

## Conclusions

Women treated by TFA with the Sonata System for symptomatic uterine fibroids reported durable improvements in generic and fibroid-specific QOL. Clinically meaningful increases in QALYs experienced at 3 years post-TFA with the Sonata System were 82% and 88% relative to perfect health when using fibroid-specific scores and 88% generic health scores, respectively.
